# Supplementary material for: Impact of 2018 ESC/ESH and 2017 ACC/AHA Hypertension Guidelines: Difference in Prevalence of White-Coat and Masked Hypertension
Source: Healthcare (Basel). 2020 May 3;8(2):122. doi: 10.3390/healthcare8020122 (PMC7348809; doi:10.3390/healthcare8020122)
Supplement: Supplementary file 1 [file healthcare-08-00122-s001.pdf]

healthcare

MDPI

Article

Impact of 2018 ESC/ESH and 2017 ACC/AHA Hypertension Guidelines: Difference in Prevalence of White-Coat and Masked Hypertension

Byong-Kyu Kim [1] and Moo-Yong Rhee [2,*]

[1] Division of Cardiology, Department of Internal Medicine, Dongguk University College of Medicine, Gyeongju Hospital, Gyeongju 38067, Korea; bleumatin@dongguk.ac.kr

[2] Cardiovascular Center, Dongguk University Ilsan Hospital, 27 Dongguk-ro, Ilsandong-gu, Goyang-si, Gyeonggi 10326, Korea

* Correspondence: mooyong.rhee@dumc.or.kr; Tel.: +82-31-961-5775; Fax: +82-31-961-7786

Received: 3 April 2020; Accepted: 30 April 2020; Published: 3 May 2020

Abstract: Our study evaluated whether there were differences in the prevalence of white-coat hypertension (WH) and masked hypertension (MH) based on the 2018 ESC/ESH and 2017 ACC/AHA hypertension guidelines in Korea. The motivation was the lowering of the diagnostic threshold for hypertension in the 2017 ACC/AHA guidelines. Of 319 participants without antihypertensive drug history and with suspected hypertension based on outpatient clinic blood pressure (BP) measured by physicians, 263 participants (51.6 ± 9.6 years; 125 men) who had valid research-grade office BP and 24-h ambulatory BP measurements were enrolled. WH prevalence based on daytime ambulatory BP among normotensive individuals was lower with the ESC/ESH guidelines than the ACC/AHA guidelines (29.0% vs. 71.4%, $p < 0.001$). However, MH prevalence based on daytime ambulatory BP among hypertensive individuals was higher based on the ESC/ESH guidelines (21.6% vs. 1.8%, $p < 0.001$). Seventy percent of WH cases (2017 ACC/AHA guidelines) and 95.2% of MH cases (2018 ESC/ESH guidelines) occurred in individuals with systolic BP of 130–139 mmHg and/or diastolic BP of 80–89 mmHg. The diagnostic threshold of the 2017 ACC/AHA guidelines yielded a higher prevalence of WH compared to that of the 2018 ESC/ESH guidelines. However, the prevalence of MH was higher with the 2018 ESC/ESH guidelines than with the 2017 ACC/AHA guidelines. The high prevalence of WH and MH in people with a systolic BP of 130–139 mmHg or diastolic BP of 80–89 mmHg suggests the need for a more active out-of-office BP measurement in this patient group.

Keywords: hypertension; guidelines; white coat hypertension; masked hypertension; ambulatory blood pressure

Supplemental tables 1–3. They were cited in the result section of the main text, consecutively.

Table S1. Degree of agreement for the diagnosis of hypertension by office measured blood pressure according to 2018 ESC/ESH and 2017 ACC/AHA hypertension guidelines. Referenced to 24-h ambulatory blood pressure.

	**Sensitivity**	**Specificity**	**PPV**	**NPV**	**Kappa**
2018 ESC/ESH guidelines	75.0	78.4	93.6	44.0	0.419
	(69.6–81.5)	(64.7–88.7)	(89.6–96.1)	(37.2–50.9)	
2017 ACC/AHA guidelines	95.9	33.3	95.2	37.3	0.308
	(92.6–98.0)	(13.3–59.0)	(93.4–96.5)	(19.6–59.2)	

PPV = positive predictive value; NPV = negative predictive value; Ref = reference; BP = blood pressure; ESC/ESH = European Society of Cardiology/European Society of Hypertension; ACC/AHA = American College of Cardiology/American Heart Association.

Table S2. Prevalence of white coat hypertension and masked hypertension based on a diagnostic threshold of the 2018 ESC/ESH and 2017 ACC/AHA hypertension guidelines. Referenced to 24 h ambulatory blood pressure.

	**Normotensives**		Hypertensive	
	Normotension n (%)	White-coat hypertension n (%)	Masked hypertension n (%)	Sustained hypertension n (%)
2018 ESC/ESH criteria	40 (78.4)	11 (21.6)	51 (24.1)	161 (75.9)
2017 ACC/AHA criteria	6 (33.3)	12 (66.7)	10 (4.1)	235 (95.9)
p value *	0.005	0.005	<0.001	<0.001

Normotensives and hypertensives were diagnosed based on 24-h ambulatory blood pressure.* *p*-value from Z-test of the difference between the rate of each phenotype of hypertension according to the criteria of the ESC/ESH and ACC/AHA hypertension guidelines. ACC/AHA = American College of Cardiology/American Heart Association; ESC/ESH = European Society of Cardiology/European Society of Hypertension.

Table S3. Prevalence of hypertension phenotypes in participants with a systolic blood pressure of 130–139 mmHg and/or diastolic blood pressure of 80–89 mmHg. Referenced to 24 h ambulatory blood pressure.

	**Normotension n (%)**	**White-Coat Hypertension n (%)**	**Masked Hypertension n (%)**	**Sustained Hypertension n (%)**
2018 ESC/ESH criteria	27 (36.0)		48 (64.0)	
2017 ACC/AHA criteria		8 (10.7)		67 (89.3)

ACC/AHA = American College of Cardiology/American Heart Association; ESC/ESH = European Society of Cardiology/European Society of Hypertension.

© 2020 by the authors. Submitted for possible open access publication under the terms and conditions of the Creative Commons Attribution (CC BY) license (http://creativecommons.org/licenses/by/4.0/).
